# Leveraging human factors in cybersecurity: an integrated methodological approach

**DOI:** 10.1007/s10111-021-00683-y

**Published:** 2021-06-11

**Authors:** Alessandro Pollini, Tiziana C. Callari, Alessandra Tedeschi, Daniele Ruscio, Luca Save, Franco Chiarugi, Davide Guerri

**Affiliations:** 1 BSD Design, Via Lazzaretto, 19, 20124 Milano, IT Italy; 2 Deep Blue Srl, Via Manin, 53, 00185 Rome, IT Italy; 3 Socio-Technical Centre, Leeds University Business School, University of Leeds, Maurice Keyworth Building, Leeds, LS2 9JT UK; 4 Dedalus, Via di Collodi, 6, 50141 Florence, IT Italy

**Keywords:** Socio-technical system, Human-centric perspective, Cyber attacks, Human error, Non-technical countermeasures

## Abstract

Computer and Information Security (CIS) is usually approached adopting a technology-centric viewpoint, where the human components of sociotechnical systems are generally considered as their weakest part, with little consideration for the end users’ cognitive characteristics, needs and motivations. This paper presents a holistic/Human Factors (HF) approach, where the individual, organisational and technological factors are investigated in pilot healthcare organisations to show how HF vulnerabilities may impact on cybersecurity risks. An overview of current challenges in relation to cybersecurity is first provided, followed by the presentation of an integrated top–down and bottom–up methodology using qualitative and quantitative research methods to assess the level of maturity of the pilot organisations with respect to their capability to face and tackle cyber threats and attacks. This approach adopts a user-centred perspective, involving both the organisations’ management and employees, The results show that a better cyber-security culture does not always correspond with more rule compliant behaviour. In addition, conflicts among cybersecurity rules and procedures may trigger human vulnerabilities. In conclusion, the integration of traditional technical solutions with guidelines to enhance CIS systems by leveraging HF in cybersecurity may lead to the adoption of non-technical countermeasures (such as user awareness) for a comprehensive and holistic way to manage cyber security in organisations.

## Introduction

The European Union Agency for Cybersecurity report “Threat Landscape 2020: Cyber Attacks.

Becoming More Sophisticated, Targeted, Widespread and Undetected” (ENISA 2020a) states that cyber-attacks have exponentially increased during the COVID-19 pandemic are expected to keep rising in number. Human and organisational factors happen to be the main contributors to, and causes of the technical and social vulnerabilities of an organisation’s Computer and Information Security (CIS) (ENISA 2020b). Additionally, businesses all over the world are going through a new wave of disruptive technological and economic changes accelerated by the recent COVID-19 pandemic. Critically, one of the significant results of COVID-19 has been the acceleration of the digital transformation of almost any commercial sector, including those that were previously more conservative (e.g. building constructions (Desruelle et al. 2019). Consequences of this transformation include: the increased number of smart workers and their delocalisation, and the subsequent transformation of the organisations’ defence perimeter; changes in the supply-chain dynamics including the supply forecasts and provisioning chains and the changes in working habit (Bicanic et al. 2020). Critically, changes of commercial, travelling and supplying habits have shortened the supply-chains (i.e., less parties involved and less geographic spread) and made them more resilient (e.g., mainly composed of trusted and resilient relationships) and less dependent by external disruptions (e.g., lockdowns) (Mudassir 2020). This *extra-ordinary* configuration has fostered cyber-crime in many weakly secured and vulnerable areas (Teal 2020).

Among the most frequent types of attacks are those that deal with Human Factor (HF) vulnerabilities (Shabut et al. 2016). These include: (1) online fraud, (2) DDoS (Distributed Denial of Service), (3) drive by download, (4) social engineering attacks (Bendovschi 2015; Katsikas et al. 2006; Sabillon et al. 2016; Shabut et al. 2016). In particular, social engineering attacks^1^ are the top threats against CIS, as they target the ‘people link’, manipulating them into divulging confidential information through influence and persuasion (Corradini 2020; Krombholz et al. 2015), or rendering sophisticated CIS technologies useless (Abawajy 2014; Dlamini et. al. 2009).

Historically, CIS has usually been approached adopting a technology-centric viewpoint, with little – if no – consideration and understanding of the end users’ cognitive processes, needs and motivations (Abawajy 2014; Aoyama et al. 2015; Glaspie 2018; Lahcen et al. 2018). As a consequence, organisations have given great emphasis to technological solutions (e.g., firewalls, antivirus software, and intrusion detection systems) to tackle potential cyber threats (Abawajy 2014; Aoyama et al. 2015; Mouton et al. 2016; Segovia et al. 2017). The recent research in cybersecurity widely agrees that a holistic approach as opposed to technical solutions alone is required to contrast cyber-attacks (Al-Darwish and Choe 2019; Bansal et al. 2010; Corradini, 2020; Jeong et al. 2019; Eminağaoğlu et al. 2009). This has been especially recognised in well-addressed sectors, such as education and healthcare, but also in novel and emerging fields, such as autonomous vehicles, where users’ behaviours and attitudes are able to undermine technological advancements (Linkov et al. 2019).

In line with this, it is argued that CIS is a systemic matter, and that a holistic/HF perspective should be taken into account to address the phenomenon (Colwill 2009; Henshel et al. 2015; Knott et al. 2013; Kraemer and Carayon 2007; Kraemer et al. 2009; Rasmussen et al. 1994; Reason 1997; Zoto et. al. 2019). The HF scientific literature has addressed the CIS phenomenon as a complex socio-technical system, in which different components interact with legitimate users to keep the system safe. Components may include organisational, technological, and environmental factors (Carayon 2006; Carayon and Kraemer 2002; Wilson 2000). Recent approaches to cybersecurity adopting a holistic socio-technical system perspective include governance and policy making issues; user-centred issues focusing on customers as well as hackers; and focused on external conditions, referred to physical, technological and economic conditions (Zimmermann and Renaud 2019). For example, social and cognitive aspects have been investigated in the healthcare cyber attacks with scenario-based simulations dealing with task- or team-centred communication, shared mental models and the availability of social support (Deline et al. 2021). Further, it has been highlighted that no matter how human-independent technology is supposed to be, eventually individuals will interface with it at various points in time (e.g., employees may happen to be in the loop when installing, configuring and maintaining technology) (Furnell and Clarke 2012; Schultz 2005). Indeed, it is recognised that the cybersecurity problem depends on the high complexity, interconnectedness and emergent qualities of socio-technical systems and that humans may be “part of the solution”, rather than “part of the problem” (Zimmermann and Renaud 2019). That is the assumption behind the non-technical countermeasures well established in literature, as opposed to ‘hard’ technical and IT security measures (e.g., Bendovschi 2015; D’Arcy and Hovav 2009; Nicho et al. 2018). Such mitigation initiatives are proposed to empower the human factor in organisations, and sustain them to be more effective against cyber-attacks and threats. As such, it is argued that humans remain a vital and inescapable element in the cyber defence of organisations, as they are critical factors in either success or failure of CIS management in organisations (Abbott et al. 2015; Eminağaoğlu et al. 2009; Glaspie and Karwowski 2018; Zimmermann and Renaud 2019).

This paper will:

1. Present an overview of the current challenges and methods related to cybersecurity;
2. Design and test an integrated method to understand and measure how healthcare organisations face the HF-related risk of cyber threats and attacks;
3. Provide an initial framework to support organisations in enhancing their CIS systems, including human factors as “part of the solution”.

## Challenges to cybersecurity

Organisations encounter a number of challenges in their effort to mitigate and/or prevent social engineering attacks (Zimmermann and Renaud 2019). The HF discipline postulates that the quality of the interdependent influences occurring between the system’s components may affect the overall human performance and actions. Should any of this interplay be weakened (e.g., poorly written rules, faulty equipment, poor management practices or unclear procedures), this can produce adverse effects, such as CIS breaches (Carayon et al. 2005; Amalberti et al. 2007; Kraemer and Carayon 2005, 2007; Kraemer et al. 2009). These can be described using a socio-technical perspective taking into account multiple perspectives, i.e., (1) the individual factors, (2) the organisational factors, (3) the technological factors and (4) the ethical dimensions.

### Challenge 1 – The individual factor

Incorrect security actions can take the form of both errors and/or violations. However, only a few of them have a malicious intent (e.g., acts of sabotage), the majority are the result of inappropriate configurations of work elements, causing accidental and non-deliberate violations, as well as deliberate actions of non-malicious intent (Rasmussen 1974, 1983; Reason 1990, 2000).

Several psychological frameworks can be used to analyse systematically individual variability related to the likelihood of error-producing conditions and violations. The Theory of Reasoned Action (Fishbein and Ajzen 1975) and the Theory of Planned Behaviour (Ajzen 1991) represent two consolidated models that link behaviours and attitudes, by the mediating effect of the so-called “behavioural intention”. According to these models, it is possible to explain human errors and violations by studying the employees' attitudes toward cybersecurity-critical behaviours, since cybersecurity can be improved attitudes predict in a direct way the actual behavioural intention of unsafe behaviours. Attitudes represent thus a crucial factor in avoiding CIS breaches related to deliberate actions determining an unwanted violation of a security rule, since cybersecurity can be improved by pushing a specific set of individual factors that are able to shape attitudes, such as subjective norms; beliefs in the perceived consequences of an action; actual knowledge of the cybersecurity topic; the preferred cognitive strategies used in a decision-making process, etc. At the same time, employees' attitudes can also enable the influence of more social and organisational factors like social norms; ethical dilemmas; and different levels of behavioural control perceived by the employee (i.e., the degree of freedom perceived to enact a given behaviour and the contextual barriers/enablers in place, related to such given behaviour). Subsequent psychological frameworks can also apply when it comes to explaining CIS breaches as violations, highlighting the role of norms and ethical values in shaping employee attitudes. According to the Norm Activation Theory (Schwarts and Howard 1984), attitudes are specifically influenced by the levels of moral obligation, self-responsabilisation, and by the explicit awareness of the consequences of a given behaviour.

Well-aware and trained employees minimise the occurrence of accidental and non-deliberate actions determining a violation of cybersecurity rules, and play a significant role in minimising information security risks and protecting the organisation’s critical assets and valuable intellectual property (Abawajy 2014; Albrechtsen 2007; Eminağaoğlu et al. 2009; Knapp et al. 2009).

Understanding the different nuances of human errors and violations can help identify the areas with the largest impact on overall system security (see in Table 1 a description of errors and violation types adapted from: Carayon et al. 2005).

**Table 1:** Taxonomy of human errors and violations

Incorrect security actions	Error/violation type	Description
Accidental and non-deliberate actions determining a violation of a security rule	Slips skill-based	Incorrect actions in tasks that are routine and require only occasional conscious checks; these errors are related to the attention of the individual performing actions relevant for security
	Lapses skill-based	Memory failures in actions relevant for security, such as omitting a planned action, losing one’s place, or forgetting security-relevant intentions
Deliberate actions determining an unwanted violation of a security rule	Rule based mistakes	Application of a bad rule relevant for security Inappropriate application of a good rule relevant for security
	Knowledge based mistakes	Intentional act involving faulty conceptual knowledge, incomplete knowledge, or incorrect action specification, leading to the unwanted violation of a security policy or procedure
Deliberate violations of a security procedure with no malicious intent	Violations	Intentional deviation from security policies or procedures due to underestimation of security consequences (can be either routine or exceptional)
Deliberate violations of a security procedure with malicious intent	Malicious violations	Intentional deviation from security policies or procedures for the purpose of sabotaging the system

### Challenge 2 – The organisational factor

Organisations have formal policies, processes and procedures to guide employees in keeping the system secure. Organisations expect their employees to be compliant with them; however, the literature has long demonstrated that formal procedures themselves do not rule human behaviour (Maalem et al. 2020). Indeed, there are many ways in which humans can configure and use a system in unexpected and/or unprotected modes and take shortcuts in the name of improving efficiency or simply being helpful, even if it implies implementing a violation (Dekker 2003; Gael et al. 2009; Schultz 2005; Stanton et al. 2005). The motivation for diverging from recommended practice may be based on informal procedures and intuitive cost–benefit evaluations where potential negative consequences of one’s act are overweighed by expected benefits (e.g., passwords that are written down or passed on to colleagues) (Besnard and Arief, 2004). Thus, when organisational policies and rules are deemed too costly, or employees do not know how to implement them in real cases, they are simply not followed Glaspie et al. 2018; Tayouri 2015). As Dekker (2003) suggests, procedures should be seen as resources for action instead of an expectation of human behaviour. Procedures must be understood: their efficiency relies more on the knowledge they require than on their blind acceptance (Besnard and Arief 2004). Albrechtsen (2007) argues that organisations are challenged to improve CIS communication to avoid possible security breaches. In his research, he reports that users see CIS as a technological discipline handled by security professionals only, complaining that ITs have poor communication with final users on correct security behaviours. Further, in relation to security documents distributed to them, the users reported: (1) lack of time to read them; (2) lack of communication on where the documentation is available; (3) lack of incentives for studying the documentation; and (4) lack of knowledge to understand CIS management instructions. He concludes that proper communication might promote the motivation of users to seek security information independently (Albrechtsen 2007).

In addition to these aspects, Da Veiga and Eloff (2010) emphasise the importance of focussing on behavioural issues by building an information security culture which embeds information security within the organisation. Indeed, a strong information security culture can contribute to minimising the risk from employee behaviour when interacting with and processing information. The security culture of an organisation reflects how management handles and treats security problems (Alhogail and Mirza 2014; Colwill 2009; Da Veiga and Eloff 2010; Da Veiga and Martins 2015). An effective CIS governance programme and policy and the quality of executive management support, as well as continuous reviews and incorporation of certain changes to meet new challenges, are all key factors in CIS effectiveness (Soomro et al. 2016). All these aspects are affected by the organisational culture need and interest and attention of the top management as they are able to impact HF-related risks of cyber threats and attacks.

### Challenge 3—The technological factor

The challenge of designing security that is effective but usable is a core aspect of the CIS phenomenon. Research has demonstrated that users actively avoid security mechanisms that are difficult to use, and/or make mistakes that might undermine security (Flechais and Sasse 2009). Security must be user-centred (Besnard and Arief 2004), but implementing user-experience principles to improve usability is still an open-issue with regard to current implementation of CIS in organisations (Flechais and Sasse 2009; Furnell et al. 2006). Poor usability in the context of cybersecurity typically translates into inadequate application of cybersecurity tools and functionality, thereby ultimately limiting their effectiveness (Nurse et al. 2011). Examples for this have been provided in the literature (Loi et al. 2019; Weber et al. 2018). Additionally, it has been argued that cybersecurity can be a hindrance to usability, particularly in relation to keeping data, systems and devices secure for vulnerable groups (e.g., Callari et al. 2012; Loi et al. 2019).

Critically, research has highlighted the challenges of incorporating individual differences and other socio-cultural variables when applying usable security design heuristics (Jaferian et al. 2011; Quiñones and Rusu 2017). Adaptive and/or personalized user interfaces have been suggested as potential ways of overcoming usability and acceptability issues related to different user domains and contexts (e.g., Addae et al. 2019).

Improvement in interface design and user experience, and it improves positive attitudes towards the correct use of that specific software and procedures (Johnston and Hale 2009). Overall, there is a unanimous agreement, that user-centric design of security products, services and policies should follow HCI principles (Carroll 2003; Shackel 2009; Sharp et al. 2007; Stanton and Young 1999) and that products designed around the users’ needs of a specific organisation in a given context, improve users’ understanding of CIS properties, and thus improving security of the systems (Besnard and Arief 2004).

### Challenge 4—Ethical dimensions in cybersecurity

Ethical questions have been a critical issue in cybersecurity (Christen et al. 2020; Macnish et al. 2020; Morrow 2018; Warren and Burmeister, 2019), and in healthcare especially (Argaw et al. 2020; Loi et al. 2019; Weber et al. 2018). With the increasing implementation of electronic healthcare information databases, if on the one hand this has improved the communication between healthcare organisations and practitioners (Coventry and Branley 2018; Yaghmaei et al. 2020), on the other, it has raised a number of concerns regarding the relationship between patients and healthcare providers and professionals and how confidentiality, integrity and availability are administered and protected (Kluge 2011; Loi et al. 2019; Weber et al. 2018). Loi and colleagues (2019) provide an overview of the relationships between the instrumental role of cybersecurity (i.e., personal data protection; Information Communication and Technology (ICT) protection; healthcare technologies/device protection) in facilitating or hindering what ICT in health aims to achieve (i.e. quality and efficiency of services; privacy; usability; and safety) and the four principles of medical ethics (i.e., (1) respect for autonomy, for patients' rights to decide for themselves regarding medical treatments; (2) non-maleficence, to reduce risks for patients deriving from medical actions/interventions; (3) Beneficence, to ensure that the best decisions are taken to improve the health status and quality of life of patients; and (4) Justice, involving the moral fairness and equality among individuals). These concern the tension that is created when, to ensure the patient's privacy and autonomy (i.e., patient password protection and encryption), critical data in emergency situations (e.g., when the patient is no longer able to agree on data accessibility, and/or when sharing the patient's data among healthcare professionals to improve the quality and efficiency of the treatment) is not accessible (Weber et al. 2018). In line with the above, Vanderhaegen (2021b) addresses the ethical dissonances that individuals may experience in different business contexts and situations (e.g., in human–machine interactions), when ethical factors are challenged by the individuals’ or groups of people’s beliefs, personal moral values and behaviours.

## Method

### An integrated methodological approach

To address the above-mentioned three CIS challenges, and to understand how organisations (and all their relevant stakeholders) could face the HF-related risk of cyber threats and attacks, an integrated approach was proposed in the context of the Horizon 2020 EU- funded HERMENEUT (Enterprises intangible Risk Management via Economic models based on simulation of modern cyber-attacks) project (HERMENEUT 2018). HERMENEUT focused on the economics of cyber security and intends to provide organisations, as well as business sectors, with an innovative methodology for the dynamic assessment of their organisational and technical vulnerabilities and the economic evaluation of the corresponding tangible and intangible assets at risk. This methodology included both the individual (i.e., considering the HF methods towards human errors) and the organisational (i.e., the role played by organisations in designing CIS-related policies and procedure on technologies) levels of analysis. The need for an integrated approach aims to go beyond the so-called “first wave” of security and privacy research on HF-related risk of cyber threats and attacks (Bødker 2006). In a recent meta-analysis, it emerged that the main focus of security and privacy researches of the last decade was mainly focused only on the individual level, i.e. considering the human actor as the primary security risk to deal with (Renaud and Flowerday 2017). However, when considering only the individual level, some well-known biases on responsibility attribution (Shaver 2012) could lead to explain cybersecurity problems only in terms of “type of user”, e.g. user with lack of cyber knowledge, lack of awareness and skills, lack of accountability, lack of reporting as well as employees with malicious intent (Zimmermann and Renaud 2019). In terms of internal validity, it means that focusing only on one level of the problem can lead the researcher to exclude alternative explanations for a given finding (e.g., influence of organisational culture) (Turner et al. 2017). A multiple-method approach can provide more complex results to handle, but at the same time it enables the triangulation of different sources for a more complex view of the phenomena (Driscoll et al. 2007) and allows for the integration of additional aspects that are as important as the individual level (Scala et al. 2019) in assessing CIS in organisations. The qualitative and the quantitative methods derived from HERMENEUT methodology and used in the present study are presented in Table 2. Priority was given to methods that allowed a quantified assessment of the level of vulnerability with respect to cyber threats, in a real-life context of different organisations involved in the research, to ensure that the actual organisational factors favouring the individual vulnerabilities could be addressed in an action research framework that could also suggest modifications to enhance real CIS systems (Ivankova and Wingo 2018).

**Table 2:** Overview of the proposed integrated method to evaluate CIS in organisations

Analysis	Objectives	Tools—Methods
Individual level		
Individual reasoning about security	Investigate the common and widespread decision-making way of thinking (heuristics and bias)	Individual interview HAIS-Q questionnaire
Accidental and non-deliberate actions determining a violation of a security rule	Investigate the causes of inadvertent human errors	Scenario- based analysis HAIS-Q questionnaire
Deliberate actions determining an unwanted violation of a security rule	Investigate the relationship between knowledge and awareness of possible source of risk	Individual interview HAIS-Q questionnaire
Deliberate violation of a security rule with no malicious intent	Investigate when and why rules are broken? Analyse the possible adaptive value of rule breaking Identify when rule breaking is required by the organisation	Individual interview HAIS-Q questionnaire
Organisational Level		
Organisation—contextual and Situational Knowledge	Organisational context: investigate human and organisational aspects as relevant areas of the enterprise dataspace Situational issues: investigate how situational variables affect the organisational performance and values	Scenario- based analysis Field observation—Contextual inquiry
Implicit rules—Modus Operandi	Investigate cultural aspects towards cybersecurity: such as salience, awareness, overconfidence	Focus group Individual interview
Explicit and formal rules	Investigate maturity towards cyber-security, describe how decisions about countermeasure are taken	Cybersecurity maturity semi-structured Interview

As presented later in Sect. 3.2, the HAIS-Q questionnaire was selected to quantify the individual level using a scientifically sound tool, while focus groups and semi-structured interviews were conducted to assess the organisational level, in line with recent qualitative research approaches (Ladner 2016; McEvoy et al. 2019). The study involved a large sample of operative roles, managers and IT experts from three different organisations working on the same domain (healthcare sector) but with different CIS systems. The first two organisations were hospitals (one from the national health system and the other from the private sector); the third organisation was a major healthcare software integrator and IT service provider. The combination of the individual and organisational levels investigated was used to produce a quantitative and qualitative evaluation aimed to build stakeholder engagement for the assessment and planning of the modification in a research-action logic.

### Research design

Overall, *n* = 94 users from the three healthcare organisations were involved in our study. Of the 94 users, 4 were managers, 32 IT experts and 58 held an operative role. Table 3 provides an overview of the approach taken in this study, and the involved users.

**Table 3:** Overview of participants and methods used for data collection

Profiles	# Participants	Method
Managers	4	HAIS-Q questionnaire
	7	Cybersecurity Maturity Semi-structured Interview
IT Experts	32	HAIS-Q questionnaire
	7	Focus Group
	5	Cybersecurity Maturity Semi-structured Interview
Operative Roles	58	HAIS-Q questionnaire
	9	Focus Group
	2	Cybersecurity Maturity Semi-structured Interview

The Human Resources (HR) departments of these organisations selected the employees to involve in the research, according to their role and representativeness (related to age, gender, background etc.). A preliminary selected group of employees received an e-mail providing the necessary information on the research and its goals, including a link to a dedicated information sheet and to a short registration form. Only the employees who registered and provided their consent were then invited to take part in the study. All participants could rely on a contact point within their organisation to receive support in case they needed additional information regarding their involvement or if they wanted to withdraw from the study before its termination. Overall, the data collection included: (1) the administration of *n* = 94 questionnaires (Sect. 3.2.1), (2) *n* = 3 focus groups involving 16 people and *n* = 14 semi-structured interviews addressed to managerial roles in the organisation (Sect. 3.2.2).

The research complied with the American Psychological Association Code of Ethics and General Data Protection Regulation (GDPR) requirements.

#### Procedure (individual level)

**Fig. 1:**
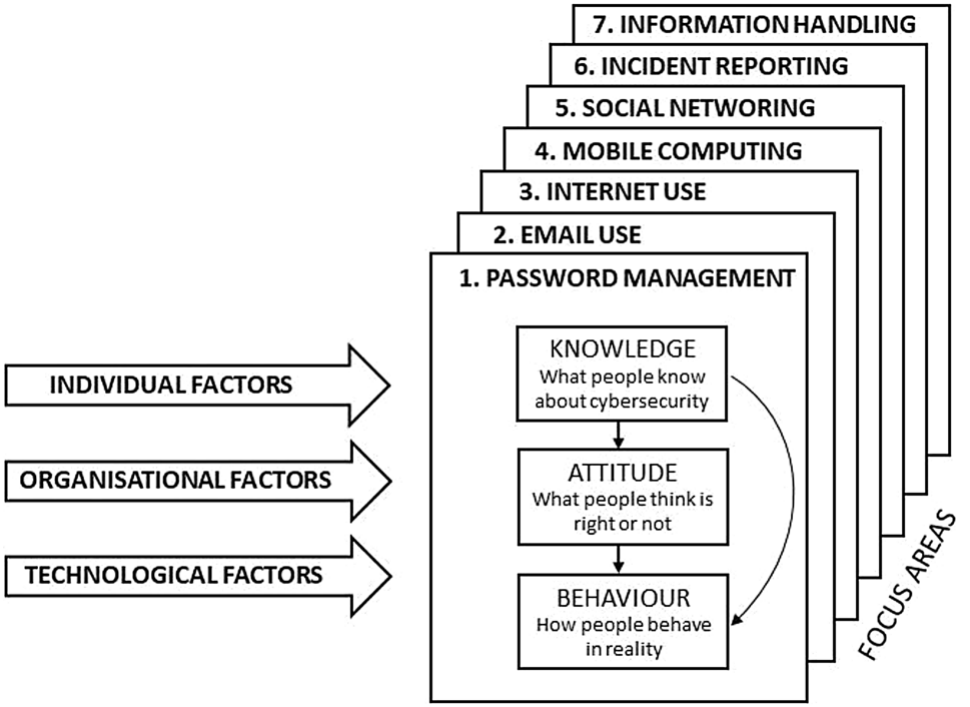
Focus areas of the HAIS-Q questionnaire

To analyse the interactions and vulnerabilities from an individual perspective, we adopted the Human Aspects of Information Security Questionnaire (HAIS – Q) (Parsons et al. 2014). In line with other recent researches (for example, Glaspie, Karwowski, 2018), the HAIS–Q questionnaire builds upon the hypothesis that as computer users’ knowledge of cybersecurity policy and procedures increases, their attitude and beliefs towards information security policy and procedures improves, which should translate into more risk-averse information security behaviour. This process is also referred to as the Knowledge-Attitude-Behaviour (KAB) model (Khan et al. 2011), as it investigates employee “Knowledge” (K) of policy and procedures; “Attitudes” (A) towards policy and Procedures and self-reported “Behaviours” (B). The HAIS-Q allows the investigation of the KAB model following seven user scenarios, henceforward ‘focus areas’ (FAs): (FA1) password management, (FA2) e-mail use, (FA3) internet use, (FA4) mobile computing, (FA5) social networking, (FA6) incident reporting and (FA7) information handling (Fig. 1).

These seven FAs resulted from findings by Parsons and colleagues and are meant to cover all the information on security policy that are relevant for employers and computer users most prone to non-compliance (Parsons et al. 2014). The HAIS-Q questionnaire presents 63 items on a 5-points Likert scale (from 1 = Strongly Agree to 5 = Strongly Disagree), divided into three batteries of items corresponding to the KAB components: the first battery includes 21 items measuring employees’ “Knowledge”; the second battery with 21 items is about employees’ “Attitude”; the third battery with 21 items is about employee self-reported “Behaviour”. Each of the 21 items within the battery covers the 7 FAs with 3 topics per FA, thus allowing the investigation of the same FA declined accordingly across the KAB components (i.e., asking three questions about the “Knowledge”, then about the corresponding “Attitude” and then about the employee actual “Behaviour” on that FA). Table 4 below presents an example of the 9 items that were used to investigate the FA1 ‘password management’ for each of the KAB components. The completion of the questionnaire took, on average, from 10 to 15 min.

**Table 4:** Example of the items concerning the topic password management

FA1—Password management
Knowledge
1. “It is possible for someone to misuse my computer if I leave it unlocked while unattended.” 2. “Personal passwords are meant for individual use only.” 3. “A strong password can be less than 10 characters long.”
Attitude
1. “I should not worry too much if I have left my computer unlocked while unattended.” 2. “It is okay to share my passwords with trustworthy people.” 3. “I believe that it is necessary for all my passwords to be at least 10 characters long”
Behaviour
1. “I lock my computer if I leave it unattended.” 2. “I share my personal password with others.” 3. “I use passwords that are at least 10 characters long.”

To prevent the risk of biasing the answers and to minimise any sequencing effect, the items were presented in a random order and were not clustered according to the KAB model. As such, it was possible to analyse the scores achieved by the items belonging to the same FA, and to compare the cases in which there was a direct/indirect correlation among KAB components.

The responses to the questionnaire were analysed with three different techniques:

- A reliability analysis of the HAIS-Q using Cronbach’s alpha
- A descriptive analysis (Mean, Standard Deviation and Median) of the sample on the KAB model components in the different FAs.
- A repeated ANOVA measure through a General Linear Model, to determine and explain the statistical differences in the sample.

For the purposes of analysis at individual level, we expected to find:

(1) A good reliability level of the questionnaire;

(2a) A direct and positive correlation among Knowledge, Attitude and Behaviour, but not necessarily in all the FAs, and

(2b) with higher absolute levels of Knowledge, compared to Attitude and Behaviours (Parsons et al. 2014);

(3a) a significant difference for highly specialised employees (i.e., IT personnel) performing better (e.g., higher HAIS-Q scores for KAB, in all FAs) than the Non-IT personnel,

(3b) especially in the IT-related organisations vs. Non-IT-related organisations.

#### Procedure (organisational level)

The focus groups and the semi-structured interviews supported the understanding in which organisational factors could potentially play a role in influencing the employees’ risk-related behaviours (McEvoy and Kowalski 2019).

##### *Focus groups*

The focus groups had the objective to provide a deeper understanding of the findings emerging from the responses to the HAIS-Q questionnaire. They involved a small sample of 16 participants among the respondents to the questionnaire. The participants were selected in a way that represented the variability of professional roles involved in healthcare: e.g., doctors, nurses, paramedics, laboratory technicians, administrative personnel and IT experts. They did not include top managers, who were recruited for the semi-structured interviews.

The focus group guideline included topics/security-scenarios derived from the FAs of the HAIS-Q questionnaire and the taxonomy of human errors and violations framework. The interpretation and analysis was carried out according to the above described taxonomy (Table 1). The sessions lasted 1.5 h in average.

##### *Semi-structured interviews*

The semi-structured interviews supported the collection of views the managers and employee representatives have in relation to the challenges faced by the organisation with respect to CIS and cyber threats, and specifically aimed to understand (i) how people make decisions about security, (ii) how they assess risk and evaluate security-critical situations, (iii) which are the most common cybersecurity policy violations. The interviews involved 14 managers and employee representatives from the three organisations. These included 2 Chief Executive Officers (CEOs), 2 Chief Financial Officers (CFOs), 3 Chief Information Security Officers (CISOs) and 7 employee representatives (namely: 5 IT experts and 2 operational roles).

The semi-structured interview protocol included the following cybersecurity topics: Governance and People, Policy and Processes, Operations, Technical controls and Attack response derived from the Cybersecurity Maturity model (Pollini et al. 2014). The interviews lasted 45 min on average.

Both the focus groups and the interviews were transcribed, and the empirical material analysed following the Thematic Analysis method (Boyatzis 1998; Braun and Clarke, 2006). The codes of the study codebook included the themes derived from the HAIS-Q questionnaires (Fig. 1), and the taxonomy of human errors and violations framework (Table 1). The codification and analysis activity were performed by two researchers to support the trustworthiness of the study findings (Nowell et al. 2017; Woods et al. 2016).

## Results

### Individual level

#### Questionnaire reliability

**Fig. 2:**
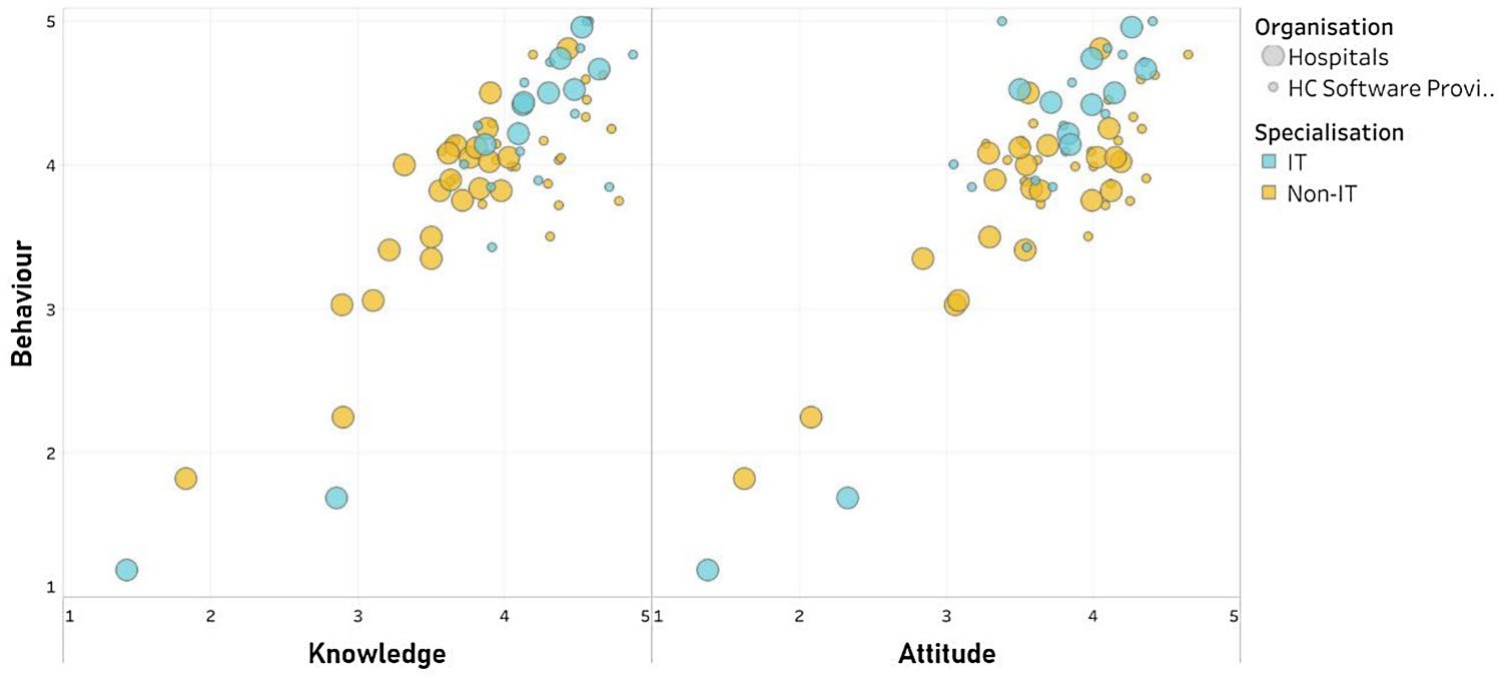
Direct, proportional and positive correlation for “Knowledge”, “Attitude” and “Behaviour” distribution across Focus Areas, for IT (blue dots) vs. Non-IT (yellow dots). The dot size represents the type of organisation: Hospitals (wider dots) vs. HC Software Provider (smaller dots)

Cronbach’s alpha provided evidence that the HAIS-Q questionnaire reached a very high degree of reliability, *α* = 0.935. This result allowed the creation of total Knowledge, Attitude and Behaviour scores as well as FA scores. The HAIS-Q questionnaire results for the entire sample reported moderate to high overall scores for the main components (Table 5), with Knowledge and Behaviour resulting in very similar levels, respectively 3.9 (0.7) and 3.9 (0.8), while Attitude score was assessed at a slightly lower level 3.7 (0.7). The overall correlations presented significant direct linear correlations between the factors Knowledge and Attitude *r* = 0.856 *p* < 0.001; Knowledge and Behaviour *r* = 0.834 *p* < 0.001; and Attitude and Behaviour *r* = 0.825 *p* < 0.001 (Fig. 2). These results are in line with previous research using HAIS-Q for employee awareness on the human aspects of information security in which the overall Knowledge is able to explain the majority of the variance in self-reported behaviour on policy and procedure (Parsons et al. 2014).

**Table 5:** Descriptive results (Overall Sample)

HAIS-Q	*N *valid	Mean	SD	Median	Percentiles		
					25%	50%	75%
Knowledge	98	3.9	0.7	3.9	3.6	3.9	4.3
Attitude	94	3.7	0.7	3.7	3.4	3.7	4.1
Behaviour	95	3.9	0.8	4.0	3.7	4.0	4.4
FA1 – password management	94	3.7	0.7	3.9	3.2	3.9	4.2
FA2 – e-mail use	94	3.9	0.8	4.1	3.6	4.1	4.6
FA3 – internet use	94	3.7	0.7	3.8	3.5	3.8	4.2
FA4 – mobile Computing	94	3.9	0.8	4.0	3.5	4.0	4.4
FA5 – social network	94	3.7	0.6	3.9	3.6	3.9	4.1
FA6 – incident Reporting	94	3.6	0.8	3.8	3.2	3.8	4.1
FA7 – information handling	94	4.2	0.9	4.3	3.8	4.3	4.8

#### IT vs. Non-IT: descriptive analysis

**Fig. 3:**
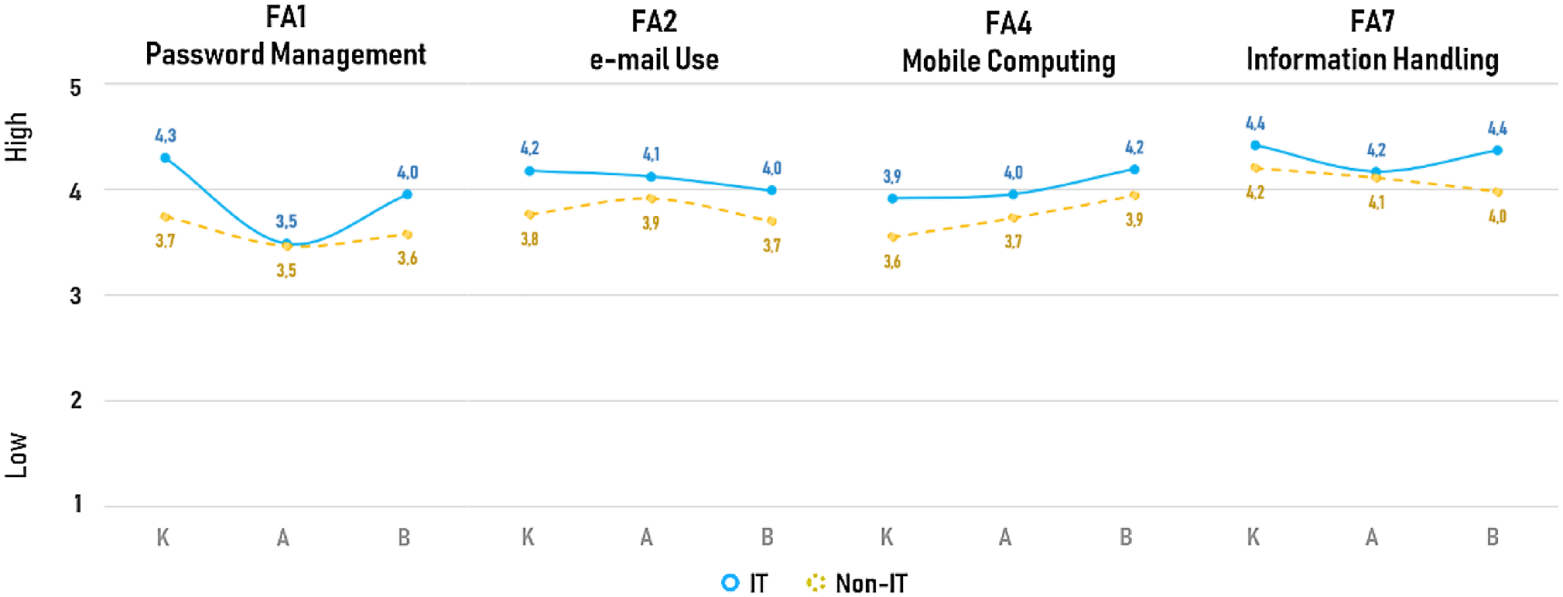
Mean Overall Scores for Knowledge (K), Attitude (A) and Behaviours (B) for the FAs where IT personnel reported total mean scores higher than Non-IT personnel

In order to better analyse the different knowledge and awareness level, attitude towards cyber-risk and reported behaviours of employees in the organisations under study, a comparison of results according to the specialisation of the respondents was carried out. When considering the different specialisation of respondents, differences were observable in various focus areas for IT personnel and Non-IT personnel, depending on the specific area investigated. For a first cluster of FAs it was possible to see an average tendency of IT personnel presenting more Knowledge, better Attitude and more correct Behaviours compared to Non-IT personnel, as one would expect. That was the case for FA1 (password management), FA2 (e-mail use), FA4 (mobile computing) and FA7 (information handling) for which IT personnel (solid blue lines in Fig. 3) reached good levels compared to a slightly more average performance of Non-IT personnel (dashed yellow lines in Fig. 3). Exceptions were represented by: the IT Attitude on FA1(password management) that reported a drop of almost 0.05 points compared to the average Knowledge and Behaviours; and FA7 (Information Handling) in which IT personnel presented a similar Attitude compared to Non-IT personnel, but still with a tendency to present more accurate Knowledge and better declared Behaviours.

**Fig. 4:**
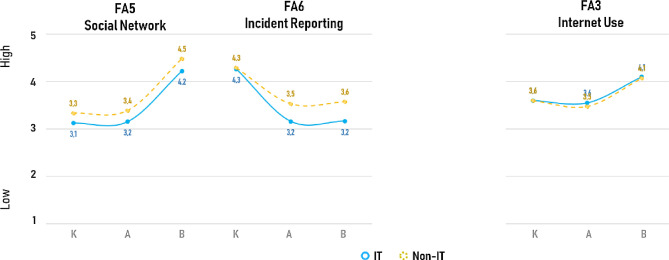
Mean Overall Scores for Knowledge (K), Attitude (A) and Behaviours (B) for the Focus Areas where IT reported total mean scores equal or lower than Non-IT personnel

On the other hand, for a second cluster of areas, the difference of IT personnel and Non-IT personnel was less evident and the overall average scores in these areas were not as high as they were in the first cluster. This was the case for Focus Areas 5 (Social Networking) in which IT personnel and Non-IT personnel presented very similar and fairly high scores for the correct self-reported Behaviours about social networks (4.2 and 4.5 scores), but with a drop of almost one point on average on the Knowledge and Attitudes in the same FA5 (Social Networking). In addition, not only did IT personnel not present good average scores on FA5, but they also scored even worse than Non-IT personnel, which presented, on average, more correct Knowledge and Attitudes towards social networks (FA5) than the IT ones (Fig. 4). Similarly, in FA6 (Incident Reporting), IT and Non-IT personnel presented an almost identically high level for Knowledge about incident report (average of 4.3 for both specialisations), but then IT personnel scored even lower scores on their Attitudes and Behaviours in comparison to the average scores reported by Non-IT personnel (Fig. 4). Finally, for the FA3 (Internet Use) the average scores of IT and Non-IT, were almost identical and not particularly high, for Knowledge and Attitudes and declared Behaviour, meaning that it was not possible to see any difference at all on the internet use of all employees, regardless of their specialisation (Fig. 4).

**Fig. 5:**
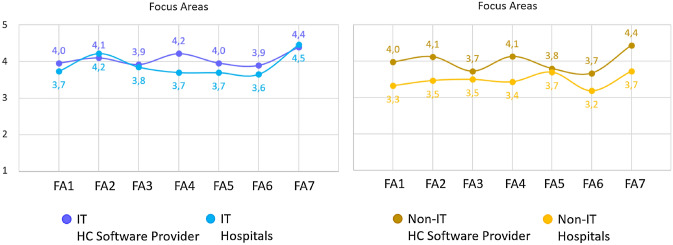
Mean Scores for the FAs for IT (blue lines, chart on the left) vs. Non-IT (yellow lines, chart on the right) personnel in different type of organisations: HC Software Provider organisations (lighter lines in both charts) vs. non-software-related organisations i.e., hospital organisations (darker lines in both charts)

The differences in the FAs also emerge as more prominent also when looking at the distribution of IT and Non-IT people in different organisations. Some organisations are able to affect the individual Knowledge, Attitude and Behaviours of their employees in a more positive way, regardless of the specialisation. As seen in Fig. 5, Non-IT scores for the personnel working in the software company are almost comparable on average to the IT scores of a non-software-related company, and clearly higher compared to the corresponding Non-IT personnel working in a hospital. Moreover, it appears that for some specific FAs, like FA4 (mobile computing) the IT specialist scores in hospitals (*M* = 3.7, SD = 0.99) are lower than the scores of Non-IT personnel working in the software company (*M* = 4.1, SD = 0.98), thus relating individual score variability also to the type of organisation in which the employees are working, and not only to their specialisation.

#### HAIS-Q statistical differences

In order to assess if the differences emerging from in the descriptive analysis reported above were statistically significant, a repeated measure ANOVA was run through a General Linear Model that considered two within dependent variables Components (3: Knowledge, Attitude, Behaviour) × Focus Areas (7) and two between-subject independent variables (2: Specialisation IT vs. Non-IT) × Organisation (2: Hospitals vs. HC Software Provider). The results confirmed that some of the differences that emerged from the descriptive analysis were also statistically significant. More precisely, the main effect for components (Knowledge vs. Attitudes vs. Behaviours) was significant F(2,94) = 24.488, *p* < 0.001, *n*_p_^2^ = 0.210, meaning that there is a significant difference between what the employees know, what the employee believes and what the employees do: these aspects do not match significantly, but they are different from individual to individual. Simple-contrast analysis highlighted that average scores in the Knowledge factor are statistically equal to the average scores in the Behaviour factor, as no significant differences between the two components were found F(1,94) = 1.791, *p* = 0.184, *n*_p_^2^ = 0.019. This suggests that the overall sample of employees are aware of what is correct and as such they behave accordingly, on average, in a safe and secure way. However, their beliefs towards policy and procedure are not perfectly in line with what they actually do, as the average score in Attitude presented low values for the whole sample F(1,94) = 38.155, *p* < 0.001, *n*_p_^2^ = 0.293. This suggests that employees' knowledge of the policies and procedures influences their behaviour more than their spontaneous attitude towards safe and secure behaviour.

The main effect of the Focus Areas was also significant: F(6,96) = 15.713, *p* < 0.001, *n*_p_^2^ = 0.146, meaning that the employees do not reach a similar level of performance in all the FAs. In some FAs the employees present very good levels of KAB, while in others the employees present significant gaps, regardless of the other variables.

This difference was even more evident in the interacting effects with the Specialisation (IT vs. Non-IT), which was also significant F(6,94) = 5.024, *p* = 0.052, *n*_p_^2^ = 0.082. IT presented statistically higher scores compared to Non-IT, as expected, in particular in overall Knowledge and Behaviours F(1,94) = 12.034, *p* = 0.001, *n*_p_^2^ = 0.116. However, this difference was not constant in all FAs. For example, when looking at the contrast analysis for IT vs Non-IT in the different FAs, a significant difference emerged in FA5 (social networking) F(1,94) = 4.628, *p* = 0.034, *n*_p_^2^ = 0.048, with Non-IT presenting higher scores than IT.

Finally, when considering the main effect of the organisation, the difference just considering the organisation alone for the whole sample (i.e., regardless of the specialisation or the FAs) was found not to be significant F(1,94) = 2.133, *p* = 0.148, *n*_p_^2^ = 0.023. However, when considering the interaction effect with the FAs and the specialisation (IT vs. Non-IT personnel), a significant effect emerged F(6,94) = 4.424, *p* < 0.001, *n*_p_^2^ = 0.047.

The Simple Contrast analysis showed that it was particularly true for two groups of FAs:

The FA3 (internet use), FA5 (social networking), and FA6 (incident reporting) where IT and Non-IT personnel scores where similar, regardless of the organisation and/or the employees’ specialisation.

This was in contrast to the FA1 (password management) F(1,94) = 8.691, *p* = 0.004, *n*_p_^2^ = 0.088 and FA7 (information handling) F(1,94) = 8.162, *p* = 0.005, *n*_p_^2^ = 0.083 where Non-IT personnel scores of software-related organisations were significantly higher compared to the Non-IT personnel of the hospital organisations, and FA4 (mobile computing) F(1,94) = 16.803, *p* < 0.001, *n*_p_^2^ = 0.157 where IT personnel scores of software-related organisations were significantly higher compared not only to the Non-IT employees, but also to IT personnel of hospital organisations.

### Organisational level

#### Focus groups

The influence of organisational factors on the knowledge, attitudes and behaviours of the employees was addressed in the focus groups, by analysing the FAs that showed a significant difference among the dependent variables being considered. The findings from the focus groups highlighted four different types of unsecure working practices that could potentially lead to cybersecurity vulnerabilities. These included: (i) the use of mobile devices (FA4), (ii) the management of access to accounts (FA1), (iii) the storing of sensitive data (FA7), (iv) the exchange of data for work coordination purposes among colleagues (FA3) and (v) communication with patients and clients (FA2).

As for the use of mobile devices (FA4), the discussion from the focus groups confirmed that Non-IT experts have less knowledge of safety risk (Fig. 3), because they tend to over-rely on the security status of mobile devices (e.g., smartphones and tablets), and therefore use them to download, manage and open sensitive attachments.

Regarding Password Management (FA1) of personal and company accounts, it was observed that the personal accounts are also used to access specific applications and/or web-based services for both private and work-related activities, whilst the latter are used for the mandatory access to company computers and medical devices. The risk associated with the combined use of different types of accounts (personal vs. company accounts) are not fully acknowledged by Non-IT personnel, thus potentially exposing the company equipment to vulnerabilities, due to the lower level of protection of the personal accounts (skill-based slips and lapses, rule-based mistakes).

As concerns the storing of sensitive data (FA7), different platforms / applications are used depending on the type of data (e.g., file servers for managing internal documents, Oracle Databases to manage personal and clinical data, GDrive to share documents with external suppliers, partners and companies). Therefore, in this case it was observed that Non-IT personnel easily get misled in storing data on platforms with remarkably different security levels and in defining suitable sharing permissions for data with very different levels of confidentiality. Similarly, private data (e.g., photographs and family documents) is stored in company PCs as a result of the increasing blurring between work and private life. Both issues derive from an inadequate perception of risks for the involved organisations also with respect to new regulations such as the General Data Protection Regulation (GDPR).

Finally, for communication (FA2) and exchange of files (FA3) it was highlighted that applications such as WhatsApp (logged in using the company computers) are sometimes used to exchange sensitive data with colleagues (e.g., pictures and scans of documents) and to coordinate work activities (e.g., shift management). These kinds of applications are also used to share clinical information with end users and/or clients, with inadequate consideration of both privacy and security requirements (knowledge-based mistakes and violations with no malicious intent).

#### Semi-structured interviews

The semi-structured interviews were useful to both identify additional sources of vulnerabilities and understand the organisational reasons behind some of the individual and organisational vulnerabilities identified with the HAIS-Q questionnaires and the focus groups. These can be framed in two main trends: (i) difficulty in managing the trade-off between cybersecurity and work efficiency, (ii) frequent risk of mismatch between the security-related restrictions imposed on the organisation’s personnel and the overconfidence of managers and IT experts regarding their personal security-related practices.

In relation to (i), the trade-off between security and efficiency manifests itself in the sharing of personal passwords and key access (FA1), such as ID fingerprints. A typical example is when doctors and nurses need to quickly access a workstation shared with a colleague. Even though knowledge of internal rules that would oblige one always to use one's own personal username and password, the time required for a logout and login procedure may result incompatible with actual behaviours in time critical tasks. In such cases, using the username and password or ID fingerprint of the colleague may not only represent the easiest way to accomplish the task, but also a concrete necessity in the interest of patient safety.

In relation to (ii), an unsecure working practice due to an inadequate trade-off between security and efficiency may result in the tendency to bypass prohibitions to install P2P software on company PCs. In specific situations, when a quick and secure solution is not available at hand, violating such implicit and explicit rules may represent the only perceived way to get the work done in an efficient manner. This may also imply paying inadequate attention to the source from which the software is downloaded and to the associated licence conditions. Another example is the need to work with an external partners (e.g., a subcontractor or third party), ensuring them with adequate connectivity to perform their work on the organisation's premises. When security policy is very restrictive and no specific arrangements have been put in place, bypass solutions (e.g., provisional Wi-Fi hotspots) can be set up to facilitate the collaboration and skip very lengthy authorization procedures. An obvious consequence of such ad-hoc arrangements is a critical reduction of the level of security. Additional unsecure practices may regard actions may be caused by the more knowledgeable IT specialists and managers in the management of cybersecurity risks. The overconfidence of IT experts in managing cybersecurity risk is typically exhibited when trying to solve specific problems requiring privileged access to company resources; this could cause new vulnerabilities or worsen existing ones. The problem can also be exacerbated by the attitude towards incident reporting (FA6), whose efficacy may be jeopardized by the persistence of forms of a blaming culture.

Even though the employees in all companies are invited to report all incidents related to security, IT experts may be reluctant to disclose incidents directly involving them, due to the erroneous perception that this could undermine their credibility. Similarly, it was observed that managers have the tendency to consider prescriptions for protecting security not directly applicable to them.

In cases such as the unsecure use of social networks (FA5), this attitude can be determined by an unintentional confusion between the restrictions required to ensure the productivity of personnel and those needed to actually protect the privacy and security of sensitive data. A blaming culture can lead to two critical consequences. The first one is a punishing and prescriptive attitude that discourages the personnel from behaving in a cooperative manner when managing security issues at an organisational level. The second one is a tendency to underestimate the negative impact of security prescriptions on the efficiency of day-by-day activities (i.e., the trade-off between cybersecurity and efficiency) and to attribute the lack of compliance with security restrictions exclusively to the personal attitude of individuals, rather than the organisational aspects of the work that would require managers to promote improvement (Chua et al. 2018).

## Discussion

The overarching aim of this study was to shed light on and deepen knowledge of the current HF challenges to contrasting cyber-attacks. To do so, it was argued that the CIS phenomenon is a systemic matter that has to be comprehended taking an HF, organisational and technical “system perspective”, in which different components interact with legitimate users to keep the system safe (Kraemer and Carayon 2007; Zimmermann and Renaud 2019). In practice this conviction does not always applied in company and business strategies aimed at building reliable organisations and cybersecurity cultures, especially when cognitive, contextual and social aspects have to be taken into account. The aim of the research was to present and discuss a systemic mixed-method approach to cybersecurity that is able to encompass human, organisational and technical countermeasures, applied to real organisations. Critically, in this systemic approach, the human factor was considered the strategic link, the ‘first line of defence’ (e.g., Parsons et al. 2017) against various information security threats; by minimising human vulnerabilities (i.e., cognitive fallacies and human errors) the organisation's security posture can be improved (e.g., Rasmussen 1983; Reason 1997).

Specifically, the objective of this study was threefold. First, it aimed to provide an overview of HF-related CIS approaches in use. Besides improving technical solutions (e.g., firewalls, implementation of encryption, etc.), we suggested integrating non-technical CIS countermeasures (Dhillon and Backhouse 2001; Jang-Jaccard et al. 2014; Siponen and Willison 2009; Siponen 2000, 2001, 2005) to improve system effectiveness (Eminağaoğlu et al. 2009). Our study confirmed this approach, as results collected from three organisations suggest that the same and most common technical solutions do not have the same impact in different working environments that have different CIS expertise and different organisational cultures.

Secondly, this study suggested an integrated method to understand and measure how organisations face the risk of cyber threats and attacks, presenting the research conducted in pilot healthcare organisations, involving different participant roles (i.e., operators and managers). This included a bottom–up and top–down approach in which both the individual and the organisational levels were involved. By doing so, a number of scenarios were investigated in a research-action approach, targeting the entire organisation whilst entering into personal experiences and work situations, to capture the motivations and intentions behind the operator's actions. The use of only one approach (Renaud and Flowerday 2017) without considering the complexity of social behaviours and their interactions with the workplaces and the technology use (Bødker 2006), would have made the explanation of some results partial and more difficult to interpret (Scaratti et al. 2017).

Thirdly, this study aimed at providing an initial framework to support organisations in enhancing their CIS systems, by including targeted guidelines for different roles for individual and organisational level assessment and support. The proposed framework integrates different analysis tools at an individual and organisational level in a sound methodology and is intended to support practitioners in the healthcare domain to timely and effectively identify human-related cyber-security vulnerabilities timely and effectively and suggest remediation measures and non-technical mitigation solutions.

At an individual level, the HAIS-Q questionnaire made it possible to investigate the extent to which individual employees’ Knowledge (K) of policy and procedures, Attitudes (A) towards policy and procedures, and self-reported Behaviours (B) were related to seven critical focus areas (FAs) of application, namely: (FA1) password management, (FA2) e-mail use, (FA3) internet use, (FA4) mobile computing, (FA5) social networking, (FA6) incident reporting and (FA7) information handling. The results showed a direct correlation between Knowledge and Behaviours, while the Attitudes towards cyber-security were more related to the different focus areas and the organisational level results. Specifically, the HAIS-Q results showed that in four focus areas Knowledge and Behaviours presented a similar trend for both IT and Non-IT personnel: FA1 (password management), FA2 (e-mail use), FA4 (mobile computing) and FA7 (information handling). With regard to password management (FA1), employers’ Knowledge and Behaviours were higher than Attitudes. This may be related to the fact that password management is a well-established and highly regulated area, and, as confirmed during the focus groups and interviews, the three organisations were indeed investing in “awareness campaigns and training” on specific CIS topics, like password management. Despite these campaigns, our research showed managers that maintaining good password management is significantly effort consuming and not always straightforward for the employees. As a result, some attitudes that could potentially become detrimental to correct Knowledge and Behaviours.

When investigating less regulated areas, like social networking (FA5) and mobile computing (FA4) or less established areas like incident reporting (FA6) and information handling (FA7), different patterns for Knowledge, Attitudes and Behaviours emerged when comparing IT and Non-IT personnel, regardless of the organisation. Not only did the IT personnel not present higher average scores on social networking (FA5) compared to Non-IT personnel, but everyone involved showed less Knowledge and Attitude toward secure use of social networks, than their actual Behaviours. This may be a consequence of the fact that they are quite new areas (i.e., mobile computing) or areas in which best practices are still underdeveloped. While for incident reporting (FA6) or secure information handling (FA7) even if the Knowledge was present, it was not matched by similar levels of Attitudes and Behaviours of the different roles (ITs and Non-ITs) within the same organisation. This could be explained by the fact that incident reporting and information handling are under the direct control and responsibility of the IT specialists, who could, therefore, underestimate the importance of reporting their own acts. At the same time Non-IT experts could be more influenced by forms of blaming culture (Craggs 2019). This aspect was carefully considered by the organisations’ management, as low scores on Knowledge or Behaviour represent potential vulnerabilities that can be exploited by a threat to trigger phishing and/or social engineering attacks.

At an organisational level, the fact that the scores of IT specialists working in the hospital organisation were significantly lower than the scores of Non-IT personnel working in HC software company, suggests that the informal organisational culture towards security can impact CIS, especially for the FAs that represent new challenges for security (e.g., FA4 Mobile Computing), where the CIS approach may not be explicitly coded (yet) into formal CIS knowledge for HC IT specialist. For instance, when mobile devices are used to download, manage and open sensitive attachments, the different sensitivity at an informal level of the software provider organisation can make a difference in terms of employee behaviour, compared to the HC organisations where the risks of mobile computing are apparently not considered in the same way.

The focus groups allowed a deeper understanding of the context (environment) and situations (events/example of critical activities) in which the areas of HAIS-Q resulted critical, also providing the rationale for their potential errors and violations. For example, when considering the common use of personal mobile devices (FA4) to access company accounts to download, store and share sensitive attachments, it was possible to understand why the organisations were exposed to CIS breaches: Non-IT experts declared they over-rely on the security of mobile devices because they did not recognize in mobile devices the same requirements necessary for using personal computers (i.e. Knowledge Based Mistakes). Conversely, Non-IT experts explained that private information was sometimes handled (FA7) using company tools and instruments, because from the user point of view, the ordinary working day “flow” can sometimes blur the boundary between the management of work and the management of private-related documents and files that they receive during the working day (i.e. Rule Based Mistakes). This suggests that more effort should be put on sensitising the employees about the actual risks posed by this of “promiscuous” information handling. Especially now that the COVID-19 pandemic has blurred even further the line between "work" and "private life”, with the increased adoption of remote working conditions, usually in private and domestic “working” environments. Additionally, a number of knowledge-based mistakes, and/or violations with no malicious intent were also highlighted in the use of technological tools and aps, such as WhatsApp (i.e., logging-in using the company computers for work purposes to exchange via the app sensitive data with colleagues or clients, and/or to coordinate work actives in an efficient manner), or as the sharing of workstations shared by different doctors and nurses (where it is common to decide to stay “logged in” with the previous user credential, because the “logout” procedure -as required by the organisation’s CIS rules- is often incompatible with the task time pressure and prioritisation of patient safety). Such everyday practices raise both privacy and security issues at an organisational level that go beyond the purely technology-centric approach to CIS.

## Conclusions

The contribution of the paper resides in the multilayered and macroergonomics methodological approach, which makes it possible to tame the complexity of human factors in Cybersecurity. The proposed approach aimed at promoting a user-centred and data-driven comprehensive and holistic approach to analysing/managing Cybersecurity in healthcare.

In particular, our study has highlighted that security countermeasures often take the form of complex procedures that provide limited support to the employees' missions and daily tasks. Our results showed that in specific FAs (e.g., Password Management, Mobile Use, Information Handling) the lack of knowledge of the correct rules and security behaviour is not the main reason for not complying with the correct security procedures. Rather, it is the result of the implicit organisational security culture that can expose the organisation to potential human vulnerabilities. We recognise the importance of highlighting the different human errors and violations as suggested by the extensive HF literature and extensive work (e.g., Reason, Rasmussen, and specifically Carayon and Kramer with a focus on CIS). However, we argue that this should be always reviewed in the context of specific users’ goals that must be achieved, and how to best achieve them. Critically, by understanding the actual activities the operators perform, and the challenges they face daily to achieve the organisational goals in their specific context of reference, one could (re)design the tool and instruments (also abiding by CIS rules) to support their work (Engestrom 2000; Lacomblez et al. 2007; Leplat 1991; Naikar et al. 2006).

In line with the literature about the CIS organisational culture (e.g., Da Veiga and Eloff 2010; Knapp et al. 2009), our results confirm that a Just Culture can help the organisations perceive the different challenges faced by their employees, and the proposed bottom–up solutions to address and overcome them (Antonsen 2009; Carroll and Quijada 2004; DeJoy 2005; Reiman and Oedewald 2007). Indeed, we agree that organisations should take formative steps to create a security-aware culture environment where security is ‘‘everyone’s responsibility’’ (Alhogail 2015). This could further support enhanced levels of understanding and trust between employer and employee with regard to the reasons for the security policies and controls that have been applied and the fact that they are in everybody’s long-term interest (Abawajy 2014).

This study supports the argument that an inclusive, multidisciplinary, holistic approach is needed to enhance cybersecurity in healthcare organisations. To understand the human vulnerabilities and the reason behind incorrect security actions taking both the form of both errors and violations, every organisation shall focus on the operator-specific needs and constraints of the work activity, to reduce the opportunities for conflicts between security and work efficiency objectives.

Therefore, a number of non-technical countermeasures are proposed to empower the human factor in organisation, and support organisations in becoming more effective against cyber-attacks and threats. This includes adopting an interwoven and user-centred design approach to promote and implement usable rules and practices, as well as fostering accountability and circulation of critical/relevant information. The following mitigation measures build a macro-ergonomic framework considered a key-factor for successful cybersecurity management when a proper integration of “technology”, “organisational policies” and “people” is achieved:

(1) When defining the core content of information security awareness programmes, assess the risk perception of employees to mitigate the perceived benefits they may foresee in risky behaviours (Glaspie et al. 2018). The perception of risks and benefits has an impact on the attitudes towards security policies and procedures, even when the knowledge of security provisions is adequate and the behaviour appears to be in line with them (e.g., Ng et al. 2009).

(2) Improve the usability of tools supporting work specific needs—such as job coordination and information sharing tasks—ensuring that their compliance with security restrictions does not jeopardize the user experience. An adequate level of user experience limits the risk of inappropriate uses of personal devices and applications with lower levels of security (Chua et al. 2018; Nurse et al. 2011). The improvement of Human Computer Interaction (HCI) -and specifically the interface design and UX interaction- will have a positive impact on the overall CIS socio-technical system, because it reduces the actual misuse of technical tools like a software or a procedure, and it improves positive attitudes towards the correct use of those specific software and procedures (Johnston and Hale 2009). Overall, there is a unanimous agreement, that user-centric design of security products, services and policies should follow HCI principles (Carroll 2003; Shackel 2009; Sharp et al. 2007; Stanton and Young, 1999) and that products designed around the specific users’ needs of a specific organisation in a given context, improve users’ grasp of CIS properties, and thus improving security of the systems (Besnard et al. 2004).

(3) When defining security policies and training campaigns, use a customised approach so that security awareness messages are commensurate to the knowledge and skills of the employees and targeted to specific information security areas (Glaspie et al. 2018). For example, consider the distinction between IT and Non-IT personnel and the difference between well-established Focus Areas—such as Password Management—and less consolidated ones, such as Social Networking (Ng et al. 2009).

(4) When designing security policies, verify the impact of the trade-offs between the security provisions and the procedures supporting other organisational goals, such as work efficiency and safety. Analyse carefully the opportunities and constraints of the working environment, as well as the needs related to the most critical tasks, to make sure that important security barriers are not bypassed just to get the work done (Woods et al. 2017). (5) Increase user motivation and knowledge, promoting a Just Culture environment in which people have an active role in improving CIS measures and are invited to provide constructive feedback on their possible limitations. A Just Culture environment implies encouraging employees to report security-related incidents, without the risk of being blamed for violations with no malicious intent (Craggs 2019).

Further studies will be needed to verify if the proposed approach can be generalized to other types of organisations, in different domains. Also, it would be interesting to consider the impact of different precursors to behaviours related to subjective and social norms, ethical factors and consequences of cybersecurity on employees in terms of emotions, (techno)stress and workload. For instance, starting from the dataset and the results of present research, it could be possible to integrate the quantitative assessment of HAIS-Q with additional standards measurement tools, to further explore -e.g., by exploratory principal component analysis- the links between the observed K-A-B variables in different FAs with and additional latent variables such as believes, ethical dissonance, technostress, burnout, individual well-being. To provide organisations with more-broader and yet agile quantitative tools to improve CIS resilience in a user-centred and data-driven way. In addition, the authors are envisioning to further extend the analysis of socio-technical systems cybersecurity further by investigating the dynamic nature of organisations as established by the active pedagogy concept (Vanderhaegen 2012, 2017, 2021a), where the technical or human components of the system are conceived as learners that autonomously evolve by accessing the required resources to act and behave according to the related norms and scenarios.

## Data Availability

Not applicable.

## Footnotes

1 Social engineering attacks refer to the “*techniques used to gain unauthorized access to information through human interaction”* (Bendovschi, 2015, p.25). Also known as human hacking *“[social engineering] is the art of tricking employees and consumers into disclosing their credentials and then using them to gain access to networks or accounts. It is a hacker’s tricky use of deception or manipulation of people’s tendency to trust, be corporative, or simply follow their desire to explore and be curious*” (Conteh and Schmick 2016, p. 31).
